# Evaluation of a remote monitoring service for patients with COVID-19 discharged from University College London Hospital

**DOI:** 10.1371/journal.pone.0284997

**Published:** 2023-07-12

**Authors:** Declan Crilly, Peter Shakeshaft, Michael Marks, Sarah Logan, Tim Cutfield

**Affiliations:** 1 Department of Infectious Diseases, University College Hospital, London, United Kingdom; 2 Information Analysis, University College Hospital, London, United Kingdom; University of Pittsburgh Medical Center Pinnacle Health Medical Services, UNITED STATES

## Abstract

**Introduction:**

In May 2020 a virtual ward for COVID-19 patients seen at University College London Hospital (UCLH) was established. The aim of this study was to see if specific factors can be used to predict the risk of deterioration and need for Emergency Department (ED) reattendance or admission.

**Methods:**

We performed a service evaluation of the COVID-19 virtual ward service at UCLH between 24/10/2020 and 12/2/2021. 649 patients were included with data collected on vital signs, basic measurements, and blood tests from their initial ED attendance, allowing calculation of ISARIC-4C mortality scores. Outcomes of interest were ED reattendance, facilitation of this by virtual ward physician, level of care if admitted, and death within 28 days of the first COVID-19 virtual ward appointment. Analysis was performed using Mann-Whitney U tests.

**Results:**

Reattendance rate to ED was 17.3% (112/649) of which 8% (51/649) were admitted. Half of ED reattendances were facilitated by the virtual ward service. Overall mortality was 0.92%. Patients who reattended ED, facilitated by the virtual ward service, had a higher mean CRP (53.63 vs 41.67 mg/L), presented to ED initially later in their COVID-19 illness (8 vs 6.5 days) and had a higher admission rate (61 vs 39%). The mean ISARIC-4C score was higher in the reattendance group compared to the non-reattendance group (3.87 vs 3.48, difference of 0.179, p = 0.003). The mean ISARIC-4C score was higher in the admission group than the non-reattendance group (5.56 vs 3.48, difference of 0.115, p = 0.003).

**Conclusion:**

Identification of patient risk factors for reattendance following a diagnosis of COVID-19 in ED can be used to design a service to safely manage patients remotely. We found that the ISARIC -4C mortality score was associated with risk of hospital admission and could be used to identify those requiring more active remote follow up.

## Introduction

The COVID-19 pandemic has placed significant strain on the NHS in the UK, particularly during surges in SARS-CoV-2 community transmission. In response, novel methods of delivering care have been required to avoid overwhelming hospital admissions. COVID-19 virtual wards are utilised to remotely manage COVID-19 patients in the community and were officially recommended by NHS England since November 2020 [1]. These services are designed to ensure safe and early discharge of COVID-19 patients from hospital.

The COVID-19 virtual ward service was set up at University College London Hospital (UCLH) following a promising preliminary study in April 2020 [2]. An evaluation of this service during September-October 2020 showed a reduction in the number of patients who had to unexpectedly reattend the UCLH Emergency Department (ED) from 22.6% to 4.7%, when comparing to rates prior to the service’s initiation [3]. This showed that virtual wards could help in managing those at risk of COVID-19 deterioration.

Since strain on healthcare services is higher when case numbers rise, we decided to evaluate the COVID-19 virtual ward service at UCLH during the Winter months of late 2020/early 2021. Assessing the service during this second wave of COVID-19 in the UK (i.e., the Alpha variant-of-concern) we were able to see if it still reduced the rate of unplanned ED reattendance.

In settings such as this, with high case numbers and finite healthcare resources, risk stratification can help identify those mostly likely to deteriorate. In other COVID-19 virtual wards, prognostic scores (such as the RECAP-V1 model) have been used to identify the highest risk patients to give them more regular follow up and/or hospital reassessment [4].

The ISARIC-4C mortality score is a prognostic score which is widely used in NHS hospitals across the UK [5]. Produced from measurements collected in most COVID-19 patients attending hospital (sex, number of comorbidities from a predefined list, Glasgow Coma Scale {GCS}, age, respiratory rate, admission oxygen saturation, urea, CRP, lymphocytes- see online tool for calculator [6]), it gives a prediction of inpatient risk of death from COVID-19 [7]. We believe it could also be used as a measure of COVID-19 deterioration risk for patients attending virtual wards.

In this study we investigated whether the COVID-19 virtual ward at UCLH was able to identify patients who were at higher risk of deterioration from COVID-19 and facilitate safe ED reattendance. We also explored whether the ISARIC-4C mortality score could be used to facilitate the identification of these higher risk COVID-19 patients.

## Methods

### Setting and study population

All adult patients with suspected SARS-CoV-2 infection, that attended UCLH ED between 24/10/2020–12/2/2021, and deemed safe for discharge, were referred to the COVID-19 virtual ward service [3]. Criteria for safe discharge from ED were oxygen saturation ≥ 94% at rest, exercise desaturation <2%, a heart rate <110 beats per minute and a respiratory rate <23 breaths per minute.

In addition to the COVID-19 virtual ward service, all patients with confirmed COVID-19 and chest X-ray changes consistent with COVID-19 and/or with persistent shortness of breath of more than 28 days after onset, were also referred to a specialist respiratory clinic for follow-up.

This study was a service evaluation of the COVID-19 virtual ward at UCLH, in examining how standard of care was delivered. It was not classed as research and so formal Health Research Authority ethics was not required.

### COVID-19 virtual ward service

Both active referrals (those made by ED and inpatient teams) and passive referrals (those with a positive COVID-19 PCR or with a coded COVID-19 diagnosis from ED) were included in the COVID-19 virtual ward service follow up. Due to limited supply, only a subset of patients was discharged with a pulse oximeter. The criteria for home pulse oximetry were CRP >50 mg/L, respiratory rate >20/min, oxygen saturations of 94–95%, or with resting oxygen saturations over 95% but with exertional desaturation of >2% (on 1 minute sit-to-stand or 40 step walk test), or typical significant COVID-19 chest X-ray abnormalities.

Lateral flow tests were not in widespread use at the time of this evaluation and therefore laboratory confirmation of COVID-19 was exclusively by upper respiratory tract SARS-CoV 2 PCR tests.

The COVID-19 virtual ward service ran 7 days a week between 09:00 to 17:00. An Infectious diseases doctor would call the patient and collect standardised clinical details on a proforma. They would then assess for risk of deterioration from COVID-19. This was based on objective elements: such as day of COVID-19 illness and oxygen saturations <94% (if they had a pulse oximeter); and subjective assessment of clinical symptoms, to form a holistic clinical impression of a patient’s risk of deterioration. A high risk of deterioration would warrant a follow up call 1 to 7 days later or, if deteriorating or requiring further investigation, a return to ED for face-to-face assessment. Patients assessed to be at low risk of deterioration would be discharged from further follow-up.

Patients were also given a leaflet with advice on infection control, self-monitoring indications for ED reattendance and the UCLH COVID-19 mobile phone number. This number allowed patients to contact a doctor 7 days between 09:00 and 17:00. Outside of these hours they were advised to call 999.

In detail description of the COVID-19 virtual ward service at UCLH can be found in S1 Fig in S1 File, taken from the previous evaluation of this service [3].

### Data collection

All data were recorded into our hospital Electronic Health Record system at UCLH: EPIC Hyperspace May 2022, Copyright ©, 2022 Epic Systems Corporation.

For each patient the following data were recorded at the time of the initial ED or inpatient episode. These include: patient’s age, gender, ethnicity, body-mass index (BMI), number of comorbidities as defined by the ISARIC-4 score, [5] C-reactive protein (CRP), total lymphocyte count, urea, day of COVID-19 illness, respiratory rate, oxygen saturation at room air, and provision of oral steroids.

The ISARIC-4C mortality scores were produced from specific patient characteristics and biochemical markers in this dataset. Because this prognostic score is widely used in the UK, validated via a UK wide consortium, and due the readily available required parameters, we used this score for this study [5].

At this time COVID-19 vaccination availability was limited and patient vaccination status records hadn’t started, so for this study this information is not available [8].

Following a patient’s initial COVID-19 virtual ward the following outcomes were recorded. Discharge from the virtual ward (including discharges with referral to the UCLH post-COVID clinic 6–8 weeks later), “facilitated” ED reattendance (defined as documented patient advice to reattend ED by a virtual ward clinician), “unfacilitated” ED reattendance (defined as no documented advice to reattend), admission to hospital, death (if within the first 28 days of the consultation), and cause of death.

For those patients that reattended UCLH ED the presentation and outcome was recorded. ED reattendances where symptoms were consistent with acute COVID-19 illness and/ or concerning complications of acute COVID-19, such as pulmonary embolus, were included. All other non-COVID-19 related reattendances were not included.

If the patient was subsequently admitted to hospital, the maximum level of care received was recorded.

### Outcomes

The primary outcome was COVID-19 associated ED reattendance or admission, within 28 days of the first COVID-19 virtual ward service appointment.

Secondary outcomes were: the ISARIC-4C score, whether reattendance was facilitated by the COVID-19 virtual ward service, and 28 days-all-cause mortality from first COVID-19 virtual ward service consultation.

### Statistical analysis

The data were collected and stored in an encrypted and password protected Excel sheet. All analyses were performed using the R Project, for Statistical Computing, programme. In the data set continuous variables were expressed as mean and standard deviation (SD) or median and interquartile range (IQR), according to their distribution. Categorical variables were described as number of total and percentage.

We used the Mann-Whitney U test to compare ISARIC-4C and patients’ hospital reattendance status. Analysis was considered statistically significant if a p value was <0.05.

### Patient and public involvement

It was not appropriate or possible to involve patients or the public in the design, or conduct, or reporting, or dissemination plans of our research

## Results

### Total cohort description

Between 24/10/2020 and 12/2/2021 there were a total of 649 patient, who received a total of 1182 virtual ward service appointments, with a median of 1 (range 1–10) appointments per patient.

The median age was 47 years (IQR 36–60). The largest ethnic group was White at 39.6% (257/ 649).

Mean CRP was 36.5 mg/L (SD: 48.1), median respiratory rate 18/min (IQR 18–20) and median oxygen saturations 95% (IQR 96–98). The median day of COVID-19 illness was 9 days (IQR 6–13 days. 5/ 649 (0.8%) patients were discharged with dexamethasone, after first ED attendance [Table 1]. All of these had been discharged from inpatient wards in late January 2021 rather than presenting to ED de novo.

**Table 1:** Demographics and clinical characteristics at ED presentation.

Patient characteristics		
Total number of patients = 649	**Median (IQR)**	**Range**
Age (years)	47 (36–60)	18–92
Respiratory rate (breaths per minute)	18 (18–20)	13–36
Oxygen saturations on room air (%)	97 (96–98)	96–98
Day of COVID-19 illness at first COVID-19 virtual ward service appointment	8.9 (5.6–12.9)	1.4–53.8
	**Mean (SD)**	**Range**
BMI (kg/m^2^)	27.7 (6)	17.1–65.8
C- reactive protein (mg/L)	36.5 (48.1)	0.6–366
Lymphocytes (x1000/μL)	1.4 (1.2)	0.11–20.3
Urea (mmol/L)	4.6 (2.17)	1.2–23.5
	**Number**	**Percentage**
Gender		
Female	317	48.8
Ethnicity		
White	257	39.6
Black	67	10.3
Asian	103	15.9
Chinese	9	1.4
Mixed	14	2.2
Unknown	126	19.4
Discharged with dexamethasone prescription	5	0.8
**COVID-19 virtual ward service**		
Total number of appointments = 1182	**Median (IQR)**	**Range**
Number of appointments per patient	1 (1–2)	1–10

### Reattendance and hospital admission rate

After an initial virtual ward service appointment, 112/ 649 (17%) patients reattended ED [Table 2].

**Table 2:** Clinical risk factors and outcomes of each of the reattendance groups.

	No ED reattendance	ED reattendance, no hospital admission	ED reattendance, hospital admission
**Clinical parameters**	**Number (%)**		
Total patient number	537 (83)	61 (9)	51 (8)
Gender			
Female	265 (49)	31 (52)	21 (41)
Ethnicity			
White	123 (23)	11 (18)	16 (31)
Non-White	137 (26)	16 (26)	14 (27)
Course of dexamethasone prescribed	4 (0.6)	1 (1.6)	0 (0)
	**Median (IQR)**		
Age (years)	47 (35–59)	46 (36–57)	59 (46.5–71)
Number of comorbidities	0 (0–1)	0 (0–1)	1 (0–2)
Day of COVID-19 illness at first appointment	9.03 (5.84–13)	7.70 (4.64–11.97)	7.48 (5.01–9.95)
	**Mean (SD)**		
C- reactive protein (mg/L)	33.95 (43.5)	42.1 (57.0)	54.2 (70.7)
Lymphocytes (x1000/μL)	1.41 (1.10)	1.41 (1.93)	0.97 (0.40)
Urea (mmol/L)	4.66 (2.26)	4.50 (1.58)	4.61 (1.91)
Oxygen saturations on room air (%)	96.97 (2.03)	97.3 (1.78)	96.1 (2.28)
BMI (kg/m^2^)	28.42 (6.13)	28.6 (5.40)	30.14 (6.22)
**Outcomes**	**Mean**		
ISARIC -4C score	3.48	3.87	5.56
	**Number**		
Deaths	3	0	3

Compared with patients that did not reattend ED, patients that reattended ED (admitted or not) had a slightly higher median age (54 vs 47 years), higher mean CRP (47.8 vs 34.0 mg/L), lower mean lymphocyte count (1.2 vs 1.4 x1000/μL) and earlier median day of COVID-19 illness (8 vs 9 days) S1 Table in S1 File.

Compared with patients that did not reattend ED, patients admitted to hospital following ED reattendance, had a higher median age (59 vs 47 years) and a higher median comorbidity number (1 vs 0 comorbidities). Ethnicity proportions were similar between these groups [Table 2].

Compared with patients who reattended ED but were not admitted, patient who were admitted had a higher mean CRP (54.2 vs 42.1 mg/L), lower lymphocyte count (0.97 vs 1.41 x1000/μL) and earlier median day of COVID-19 illness (7 vs 8 days) at time of initial COVID-19 assessment. One of the 5 patients who were prescribed dexamethasone after initial ward discharge reattended ED [Table 2].

Compared to patients with unfacilitated ED reattendances, patients with facilitated reattendances had a higher mean CRP (53.63 vs 41.67 mg/L), later median day of COVID-19 illness (8 vs 7 days) and higher percentage likelihood of being non-white ethnicity (32% vs 21%). A higher number of patients with facilitated reattendance were admitted to hospital (31, 60%) compared with patients with unfacilitated reattendance (20, 39%). The above and the level of care data is available in S2 Table in S1 File.

### Mortality

There were 6 deaths within this cohort (0.92%). All deaths occurred in hospital, although 3 were admitted to hospitals other than UCLH. These patients were all over 50 years of age, with comorbidities, and were managed with at least non-invasive ventilation. All deaths were attributed to/ related to COVID-19.

### ISARIC-4C score

**Fig 1:**
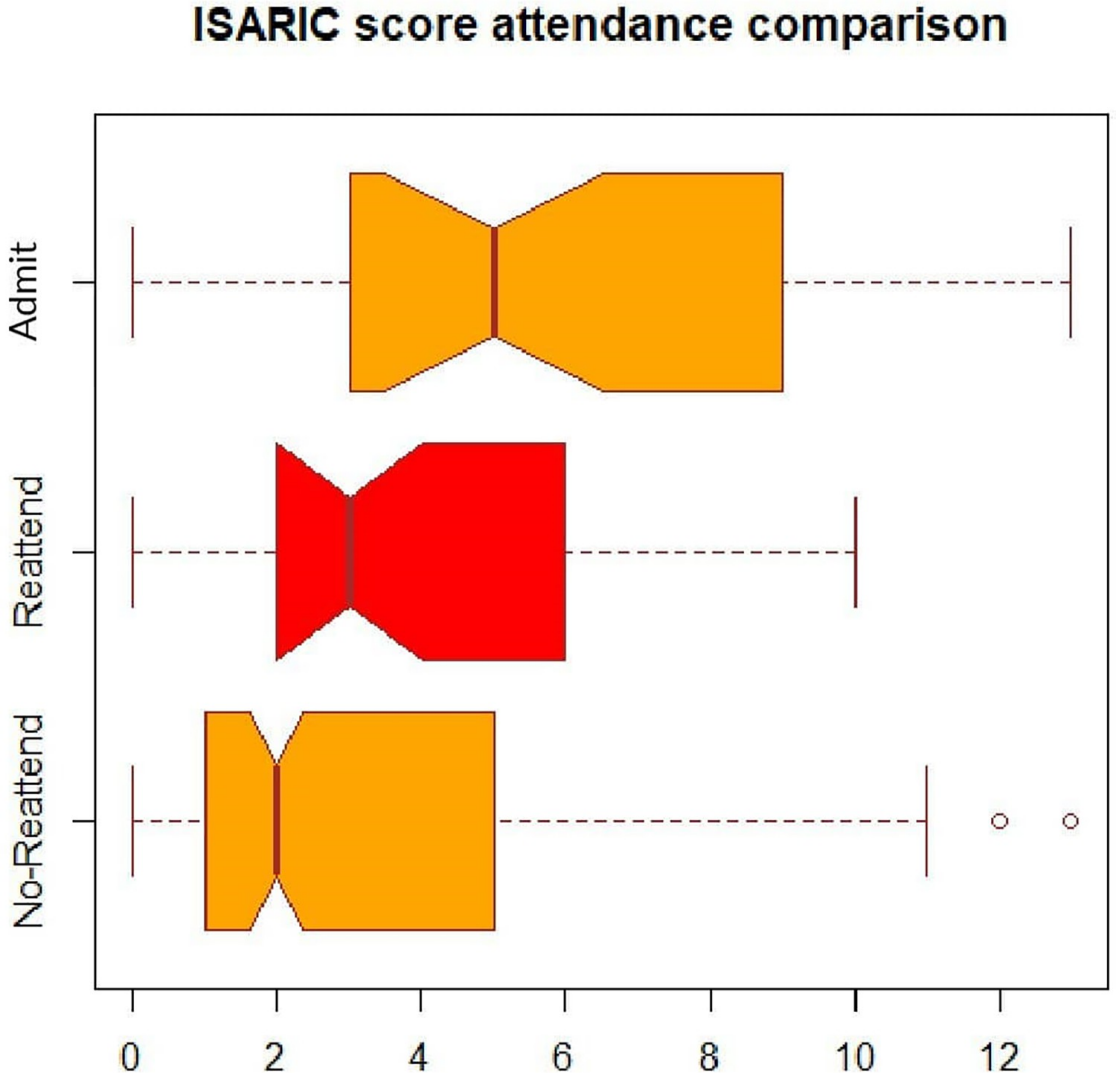
A figure showing the comparison between ISARIC- 4C mortality scores of non-reattender, reattender and admitted patients.

Mean ISARIC-4C score was significantly higher in patients who reattended ED than patients who did not reattend ED (3.87 vs 3.48, p = 0.003). It was also significantly higher in patients who were admitted to hospital after ED reattendance than patients who did not reattend ED (5.56 vs 3.48, p = 0.04) [Fig 1].

There was no significant difference between the ISARIC-4C score of unfacilitated and facilitated reattendances (4.82 vs 4.60, p = 1).

### Comparison within our service

Compared to previous service evaluations performed in July [2] and September [3] 2020, patients in this evaluation had a higher mean CRP (18.8 vs 8.4 vs 2.2mg/L), a higher number re-attended ED (112 vs 12 vs 32) and were more admitted to hospital (51 vs 5 vs 7) [Table 3].

**Table 3:** Comparison of cohort demographics, clinical characteristics, and outcomes.

	27/4/2020-3/6/2020, [2]	1/9/2020-23/10/2020, [3]	24/10/2020–12/2/2021
**Clinical parameters**	**Number**		
Total patient number	192	134	649
	**Number (%)**		
Gender			
Female	96 (50)	68 (50.7)	317 (48.8)
	**Median (IQR)**		
Age (years)	43 (32–55)	41 (28–56)	47 (36–60)
Oxygen saturations on room air (%)	98 (97–99)	97.5 (96–99)	97 (98–96)
C- reactive protein (mg/L)	2.2 (0.7–11.0)	8.4 (1.3–25.4)	18.8 (6.2–46.6)
**Outcomes**	**Number (%)**		
Total reattendances	32 (16)	12 (9)	112 (17.3)
Facilitated reattendances	23 (12)	5 (3.7)	56 (8.6)
Unfacilitated reattendances	9 (4.7)	7 (5.2)	56 (8.6)
Total admissions	7 (3.6)	5 (3.7)	51 (7.9)
Facilitated admissions	5 (2.6)	2 (1.5)	31 (4.8)
Unfacilitated admissions	2 (1)	3 (2.2)	20 (3.1)
28-day mortality	0(0)	0(0)	6 (0.92%)

There were no deaths in previous cohorts.

## Discussion

In this study we found that risk stratification of COVID-19 patients attending virtual wards, via subjective clinical assessment and objective ISARIC-4C mortality scores, was beneficial in identifying those most likely to deteriorate from COVID-19.

We observed that those patients with more risk factors for disease progression and evidence of more severe COVID-19, i.e. older, comorbid, higher CRP, lower lymphocyte count, low oxygen saturations and earlier day of COVID-19 illness, [9, 10] were more likely to require ED reattendance.

Additionally, we have shown that there is a difference between the ISARIC-4C scores of ED reattenders and non-reattenders, so that it can be used to help identify those more likely to deteriorate with COVID-19.

In this study the ISARIC- 4C mortality score was used to determine risk of deterioration, rather than the ISARIC -4C deterioration score, due to the availability of required parameters. We have shown that there were statistically significant differences between the groups in our cohort however, there was significant overlap between these groups. It is plausible that the ISARIC -4C deterioration score may have offered an improved discrimination of risk [11]. As developed and validated prior to widespread COVID-19 vaccination availability, further investigation into these prognostic scores current applicability is warranted. As seen with the lack of difference between unfacilitated and facilitated reattendances in this study, there may have been patients who deteriorated more than expected or who reattended without recommendation. This highlights the importance of close patient evaluation on a case-by-case basis to assess risk of deterioration, and not just relying on risk stratification tools.

We noted a higher number of ED reattendances, hospital admissions and deaths in this study compared with earlier analyses of the UCLH virtual ward service [Table 3] [3]. Extrapolation of these results may be difficult given the different cohort sizes, but they are likely explained by the prevalence of more clinically severe disease, evidenced by higher mean CRP and lower mean oxygen saturations in this cohort.

The difference in reattendance rate likely reflects a change in management in response to the worsening clinical severity seen at this point in the COVID-19 pandemic. During the surge of cases in Winter 2020, unprecedented pressures were placed on inpatient hospital beds [12]. At UCLH, as elsewhere, the raised threshold for hospitalisation meant more unwell patients were discharged into the care of the COVID-19 virtual ward service. This may explain the higher ED reattendance and mortality rates, compared to previous cohorts. This increase in disease severity is reflected in other UK COVID-19 virtual wards, which showed comparable ED reattendance rates (4–36%) and mortality rates (0–29%) [13].

This UCLH COVID virtual ward service evaluation is part of a growing literature on a subject that has expanded enormously since the pandemic began. Due to their essential, but rapid development, understanding and measuring the benefit of virtual wards has faced many challenges and produces mixed results.

In a similar timeframe to this study, a retrospective single centre study of 43 patients at a virtual ward of a UK district general hospital (January—March 2021), showed a reduced mean length of hospital stay for COVID-19 virtual ward attendees (10.3 days +/- 9.7 days) compared to all COVID-19 positive patients at this hospital (11.9 days +/- 11.6 days) [14]. Moreover only 9% required admission, like admission rates in these systematic review papers [13, 15]. These review papers also report good clinical outcomes for COVID-19 virtual wards. Published on the 26^th^ May 2021, a systematic review of 6 databases across 7 countries on COVID-19 virtual wards reported mortality rates of 0–3.1%, reattendance rate of 4–36% and admission rates between 0–29% [13]. A multi-site mixed qualitative and quantitative methods study between July and August 2020 of 8 study sites (looking at 1737 patients) found a similarly low mortality rate (1%) [15].

However, neither of these studies were able to confidently assess the effectiveness of virtual wards, due to lack of standardised reporting and control groups. Limited exploration of virtual wards’ cost-effectiveness and resource allocation, considering the concurrent non-COVID-19 workload outside of the initial stages of the pandemic, may limit their utility [13, 15].

The provision of home oximeters has often been promoted as a core aspect of COVID-19 virtual ward services, [16] however one study indicated pulse oximetry results do not always accurately reflect arterial oxygen saturation [17]. Moreover a multivariate analysis from national observational data of 123 English hospital trusts between August-February 2021 showed where COVID-19 virtual wards were available, inpatient stays were 5% longer. The authors hypothesise that this may be related to earlier admissions and improved bed capacity from simultaneous virtual ward set up, at the tail-end of the UK’s second wave [18].

In our study, although pulse oximeters were given to patients fulfilling specific ‘high risk’ criteria, the data was not available and so cannot comment on their effectiveness. We believe that oximetry is probably still useful in remote clinical assessment, for normal oxygen saturations in mild COVID-19 cases may be reassuring enough to not require ED reattendance.

The COVID-19 vaccination status of patients was not included in the study, as it was not available from the clinical records. At the time of the study only a small proportion of the UK population would have received a COVID-19 vaccine. These would have been frontline healthcare and social workers, or those with increased risk of serious disease or death from COVID-19 (i.e. organ transplant recipients, those with specific cancers, the immunosuppressed, those with severe chronic respiratory conditions or significant congenital diseases) [8]. Even within this cohort the 12 week gap between COVID-19 vaccine doses recommended at the time, meant that most would’ve only had a single dose [19]. It’s lack of inclusion in the study, therefore, may not have affected results. The widespread COVID-19 vaccination status (including boosters) of the population, with the Omicron and Delta variants and the emphasis on the importance of mass vaccination [20], means it should now be included in further evaluations to assess impact on deterioration risk [21].

Limited data also prevented accurate evaluation of level of care received on admission and discharge dexamethasone provision (as well as other outpatient treatments), so these should be studied further.

When incorporating the experiences of our study with that of healthcare services in the UK, and internationally with other similar risk stratification tools, it is important that the differences in COVID variants, community prevalence, access to COVID-19 testing, outpatient COVID-19 treatments, and access to COVID-19 vaccination are all considered when building on COVID-19 virtual wards. When evaluating COVID-19 virtual wards it is important that an assessment of their clinical effectiveness, impact on healthcare systems and cost must be made and properly evaluated, to best adapt them for the contemporary phase of the COVID-19 pandemic.

## Conclusion

The COVID-19 virtual ward service at UCLH identified patients with demographic and clinical risk factors associated with increased risk of deterioration. The ISARIC-4C score may be a useful additional method of stratifying risk that might improve the focussed management patients at highest risk of clinical deterioration.

COVID-19 virtual ward services have helped to safely manage patients outside of the hospital setting in the UK prior to widespread vaccination. Prospective studies are needed to better define patient groups likely to benefit from COVID-19 virtual support in contemporary and future phases of the pandemic.

## Supporting information

S1 File (PDF)
