# Supplementary material for: Evaluation of a remote monitoring service for patients with COVID-19 discharged from University College London Hospital
Source: PLoS One. 2023 Jul 12;18(7):e0284997. doi: 10.1371/journal.pone.0284997 (PMC10337886; doi:10.1371/journal.pone.0284997)
Supplement: S1 File — (PDF) [file pone.0284997.s001.pdf]

S1 File. Supporting Information

Figure A. COVID- 19 virtual ward SOP at UCLH

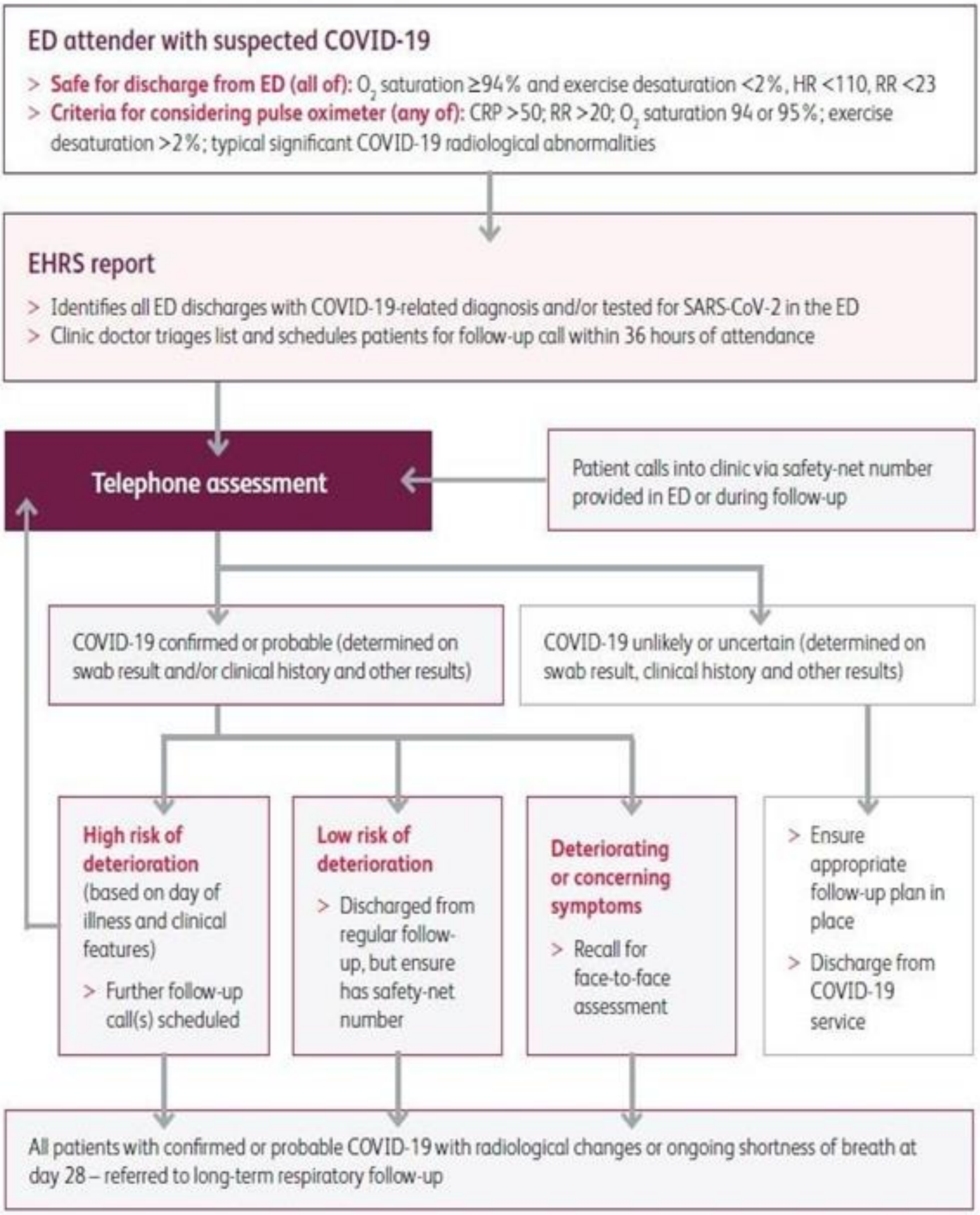

Fig 1. Rapid remote follow-up pathway. CRP = C-reactive protein; ED = emergency department; HR = heart rate; RR = respiration rate.

Table A - Comparison between risk factors and outcomes of total reattendance and non-reattendance groups

	**No ED reattendance**	**ED reattendance**
**Clinical parameters**	**Number (%)**	
Total patient number	537 (83)	112 (17)
Gender<br>Female	 265 (49)	 52 (46)
Ethnicity<br>White<br>Non-White	 123 (23)<br>137 (26)	 27 (24)<br>30 (27)
Course of dexamethasone prescribed	4 (0.6)	1 (1.6)
	**Median (IQR)**	
Age (years)	47 (35-59)	54 (40-64)
Number of comorbidities	0 (0-1)	0 (0 -1)
Day of COVID-19 illness at first appointment	9.03 (5.84-13)	7.53 (4.91- 10.92)
	**Mean (SD)**	
C- reactive protein (mg/L)	33.95 (43.5)	47.8 (63.8)
Lymphocytes (x1000/µL)	1.41 (1.10)	1.21 (1.45)
Urea (mmol/L)	4.66 (2.26)	4.55 (1.74)
Oxygen saturations on room air (%)	96.97 (2.03)	96.75 (2.10)
BMI (kg/m^2)	28.42 (6.13)	29.57 (5.93)
**Outcomes**	**Mean**	
ISARIC -4C score	3.48	4.70
	**Number**	
Deaths	3	3

Table B - Comparison between risk factors and outcomes of facilitated and unfacilitated reattendances

	**Facilitated reattendance**	**Unfacilitated reattendance**
**Total numbers (n, %)**	56 (50%)	56 (50%)
**Age (Median, IQR)**	50, 62.25- 42.50	55.50, 68.25- 38.25
**Gender -Female (n, %)**	23 (41%)	23 (41%)
**Ethnicity (n, %)**	White= 15 (27%)<br>Non white= 12 (21%)	White= 12 (21%)<br>Non white= 18 (32%)
**BMI (n, %, mean, SD)**	36 (64%)<br>30.95, 6.44	36 (64%)<br>28.18, 5.10
**Comorbidities (median, IQR)**	0, 1-0	0, 1-0
**Last CRP (mean, sd)**	53.63, 70.97	41.67, 55.37
**Lymphs (mean, sd)**	1.09, 0.48	1.34, 2.02
**Urea (mean, sd)**	4.45, 1.51	4.68, 1.99
**Sats (mean, sd)**	96.7, 1.70	96.9, 2.47
**Day of illness at first CRAID (median IQR)**	7.95, 11.06- 5.91	6.61, 10.85- 3.61
**Readmitted (n,%)**<br>**Total= 51**	31 (61%)	20 (39%)
**Level of hospital care (n,%), of those readmitted**	Ward = 29 (56%)<br>CPAP = 5 (10%)<br>Intubated = 2 (4%)<br>ECMO = 0	Ward = 24 (47%)<br>CPAP = 0<br>Intubated = 2 (4%)<br>ECMO = 1 (2%)
**Death (n, %)**	2 (4%)	1 (2%)
