# Supplementary material for: Evaluation of a remote monitoring service for patients with COVID-19 discharged from University College London Hospital
Source: PLoS One. 2023 Jul 12;18(7):e0284997. doi: 10.1371/journal.pone.0284997 (PMC10337886; doi:10.1371/journal.pone.0284997)
Supplement: Attachment — Submitted filename: Response to Reviewers.docx [file pone.0284997.s002.docx]

**PLOS One Journal Editorial Office**
The Bradfield Centre,

184 Cambridge Science Park

Milton, Cambridge, CB4 0GA
United Kingdom

Email: [plosone@plog.org](mailto:plosone@plog.org)

Tel: +44 (0)1223442810

Dear PLOS One reviewers

RE:	*Evaluation of a remote monitoring service for patients with COVID-19 discharged from University College London Hospital*

Many thanks for your kind consideration of this manuscript for publication in the PLOS One Journal, and thank you for the thoughtful comments regarding changes and alterations to the manuscript.

I have made the changes (tracked and in full) in the 2 uploaded manuscript documents.

I appreciate the suggestion for the inclusion of the article by Lokesh Goyal et al, and have included its reference in the Discussion. It solidifies the studies relevancy to current times with regards to vaccines, boosters and their necessity for control of current COVID-19 variants.

I have also included a brief description of the ISARIC-4C calculator parameters and a link to the calculator website ( <https://isaric4c.net/risk/>) in the Introduction as it seemed most appropriate at this point as it was/is widely used in all UK hospitals for COVID-19 inpatient mortality risk.

I have edited and made the grammatical and format changes to the rest of the article, hopefully to satisfaction.

Please let me know if there are any further suggestions you would make regarding the manuscript and thank you once again for reviewing this article.

Kind regards,

Yours sincerely,

Dr Declan Crilly and the UCLH COVID-19 virtual ward team

Infectious Diseases SHO and General Medical Registrar

University College Hospital London, UK

07972091424 -Email: [declan.crilly@nhs.net](mailto:declan.crilly@nhs.net)
