# Supplementary material for: Evaluation of a remote monitoring service for patients with COVID-19 discharged from University College London Hospital
Source: PLoS One. 2023 Jul 12;18(7):e0284997. doi: 10.1371/journal.pone.0284997 (PMC10337886; doi:10.1371/journal.pone.0284997)
Supplement: Attachment — Submitted filename: Response to Reviewers 16.2.docx [file pone.0284997.s003.docx]

**PLOS One Journal Editorial Office**
The Bradfield Centre,

184 Cambridge Science Park

Milton, Cambridge, CB4 0GA
United Kingdom

Email: [plosone@plog.org](mailto:plosone@plog.org)

Tel: +44 (0)1223442810

Dear PLOS One reviewers

RE:	*Evaluation of a remote monitoring service for patients with COVID-19 discharged from University College London Hospital*

Many thanks for your kind consideration of this manuscript for publication in the PLOS One Journal, and thank you for the thoughtful comments regarding changes and alterations to the manuscript.

I have made the changes (tracked and in full) in the 2 uploaded manuscript documents.

I have hopefully clarified the aspects relating to: dexamethasone provision in a small number of patients on initial attendance to ED, comparison results of ED non reattendance vs ED reattendance (combining admission and non-admission groups), as well as the importance of using physician clinical acumen on a case by case basis particularly considering the lack of difference between ISARIC-4C scores of facilitated and unfacilitated ED reattendance groups.

I had wanted to clarify where you felt insufficient data had been provided for interpreting the study, so that I may alter that aspect?

Please let me know if there are any further suggestions you would make regarding the manuscript and thank you once again for reviewing this article.

Kind regards,

Yours sincerely,

Dr Declan Crilly and the UCLH COVID-19 virtual ward team

Infectious Diseases SHO and General Medical Registrar

University College Hospital London, UK

07972091424 -Email: [declan.crilly@nhs.net](mailto:declan.crilly@nhs.net)
